# Spontaneous Tumor Lysis Syndrome in a Patient With a Known History of Chronic Lymphocytic Leukemia Prior to Cytotoxic Chemotherapy

**DOI:** 10.7759/cureus.36305

**Published:** 2023-03-17

**Authors:** Marcus Lee, Michael Sandhu

**Affiliations:** 1 Department of Medicine, Stony Brook University, Stony Brook, USA; 2 Department of Internal Medicine, State University of New York (SUNY) Upstate Medical University, Syracuse, USA

**Keywords:** rasburicase, leukocytosis, electrolytes, chronic lymphocytic leukemia, spontaneous tumor lysis syndrome

## Abstract

Tumor lysis syndrome (TLS) is an oncological emergency resulting in an imbalance of electrolytes released upon tumor cell death leading to life-threatening acute renal failure. Typically, TLS is triggered by cytotoxic chemotherapy; however, it can rarely occur spontaneously. Our case report presents a patient with a known malignancy, but not on any cytotoxic chemotherapy, who presents to the emergency department with metabolic derangements suggestive of spontaneous TLS. Our case highlights the importance of considering an uncommon manifestation of TLS despite the absence of cytotoxic chemotherapy. This case is unique as it demonstrates the manifestations of TLS in a patient with a known stable malignancy and discusses the subsequent management.

## Introduction

Chronic lymphocytic leukemia (CLL) was the most common leukemia diagnosed in adults in the United States in 2021, comprising roughly 34.9% of leukemias diagnosed [1]. According to the International Workshop on Chronic Lymphocytic Leukemia (iwCLL), the diagnosis of CLL requires a lymphocyte count of ≥5 × 10^9^/L for at least three months [2]. The lymphocytes on peripheral blood smear appear as “characteristically small, mature lymphocytes with a narrow border of cytoplasm and a dense nucleus lacking discernable nucleoli and partially aggregated chromatin” [2]. CLL can present with anemia, thrombocytopenia, splenomegaly, lymphadenopathy, progressive lymphocytosis (defined as a lymphocyte doubling time of <6 months), and autoimmune complications. Patients may also have unintentional weight loss of ≥10% within six months, fatigue, and fevers to ≥38°C or night sweats for ≥1 month without evidence of infection [2].

Tumor lysis syndrome (TLS) is an oncological emergency resulting in an imbalance of electrolytes released upon tumor cell death leading to life-threatening arrhythmias and acute renal failure [3]. Typically, TLS is triggered by cytotoxic chemotherapy; however, it can rarely occur spontaneously [3]. Our case report presents a patient with a known malignancy, but not on any cytotoxic chemotherapy, who presents to the emergency department with metabolic derangements consistent with spontaneous TLS.

## Case presentation

An 85-year-old male with a known diagnosis of CLL initially presented to an outside hospital for two weeks of progressive shortness of breath, chest pain, and abdominal distension. The patient did not report any other symptoms. The patient had been on surveillance for the management of his CLL without any treatment. He was following up with his oncologist for this. He had imaging five months prior to presentation, which showed moderate cervical and axillary adenopathy, but was stable from the previous imaging obtained two years ago. His white blood cell (WBC) count was consistently around 111,000 cells/μL for the past six months prior to presentation. His prior flow cytometry showed CD5+ clonal B cells (7%) with increased monocytic cells (18%) with partial aberrant CD56 expression, but no increase in CD34+ blasts.

At the outside hospital, cardiology was consulted, and a 2D echocardiogram was done, which showed a large pericardial effusion with evidence of tamponade physiology. From outside records, the patient’s echocardiogram performed six months prior showed normal LV dysfunction with mild mitral regurgitation, but no significant valvular disease. Blood testing performed at the outside hospital reportedly showed hyperkalemia, high uric acid, and leukocytosis of 189,600 cells/μL. The patient was then transferred to our facility for a higher level of care.

On admission, the patient appeared to be short of breath. He also complained of rhinorrhea but denied any fever, cough, or chest pain. The patient’s vital signs were stable, and he did not require supplemental oxygen. Physical examination showed an irregular heart rhythm and distant heart sounds as well as cervical lymphadenopathy. Peripheral blood smear showed lymphocytes increased in number with a mature appearance consistent with CLL.

**Figure 1:**
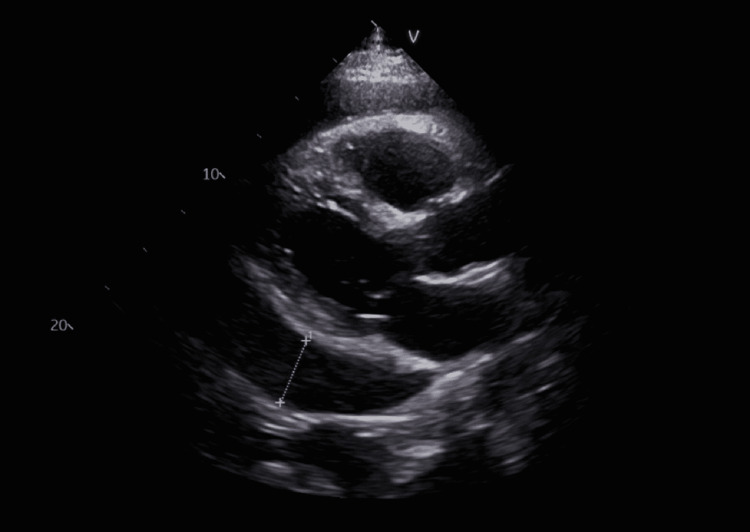
Echocardiogram: parasternal long-axis view White line: pericardial effusion

**Figure 2:**
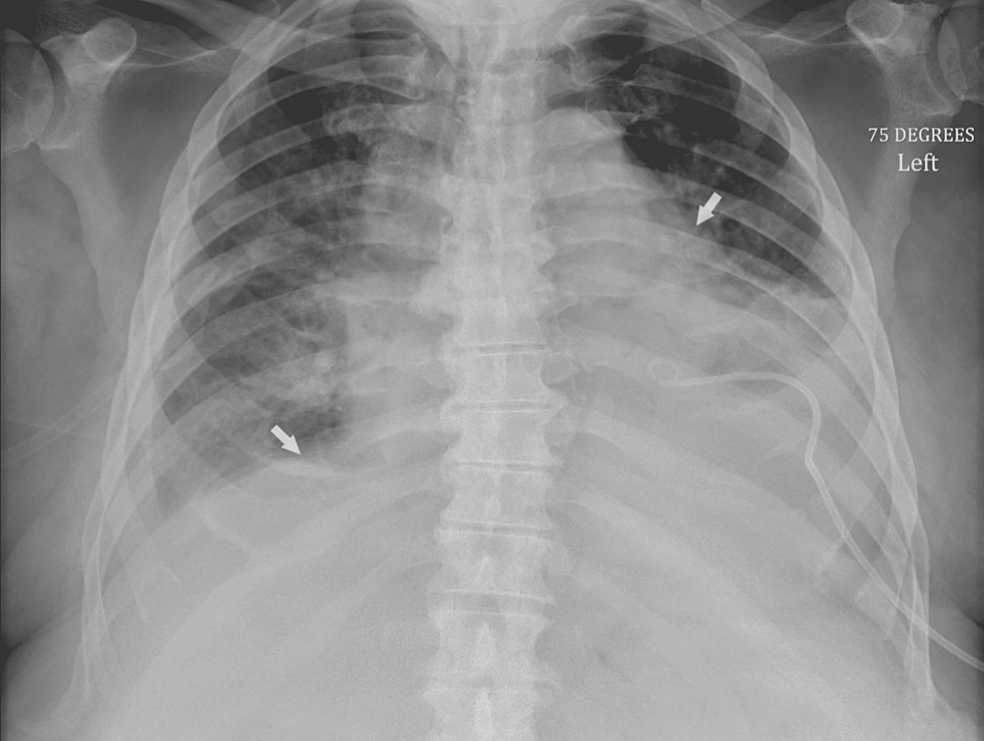
Chest X-ray showing the bilateral pleural effusion White arrows: bilateral pleural effusion

The patient’s laboratory values on admission are shown in Table 1. Repeat evaluation of serum potassium the following day resulted in 5.5 mmol/L. Repeat 2D echocardiogram confirmed the pericardial effusion, as shown in Figure 1, and showed a left ventricular ejection fraction of 58%. Chest X-ray showed cardiomegaly with pulmonary vascular congestion and bilateral pleural effusions, as shown in Figure 2. Given the elevated WBC counts, hyperkalemia, hyperphosphatemia, and high-normal lactate dehydrogenase levels, there was a concern for spontaneous tumor lysis syndrome (Table 2).

**Table 1:** Laboratory investigations on patient’s admission and their respective values The laboratory values shown are during the day of the patient’s presentation. His prior creatinine one year ago was 1.59 mg/dL.

Laboratory investigations	Laboratory values	Reference range
Hemoglobin (g/dL)	6.3	13.5-18
Hematocrit (%)	21.1	41-53
Platelet count (cells/uL)	108,000	150,000-400,000
White blood cell count (cells/uL)	165,900	4,000-10,000
White blood cell differential		
Neutrophils (%)	14	40-60
Lymphocytes (%)	78	20-40
Monocytes (%)	8	2-8
Potassium (mmol/L)	5.1	3.4-5.1
Creatinine (mg/dL)	2.65	0.70-1.20
Calcium (mg/dL)	8.7	8.8-10.2
Phosphorous (mg/dL)	6.7	2.5-4.5
Albumin (g/dL)	3.6	3.5-5.2
Uric acid (mg/dL)	9	3.4-7
Lactate dehydrogenase (U/L)	218	122-225

**Table 2:** Cairo-Bishop classification of tumor lysis syndrome TLS: tumor lysis syndrome, AKI: acute kidney injury

Laboratory TLS	Clinical TLS
Uric acid > 8 mg/dL	AKI (defined as creatinine > 1.5× the upper limit of normal for patient age and sex)
Potassium > 6 mEq/dL	Cardiac arrhythmia
Phosphorus > 4.6 mg/dL	Seizure, tetany, or other symptomatic hypocalcemia
Calcium < 7 mg/dL	

The patient underwent a pericardiocentesis that drained approximately 1 L of fluid, and a pericardial drain was placed. The pericardial fluid was sent for cytological analysis and was positive for malignancy, consistent with chronic lymphocytic leukemias. Oncology was consulted and recommended starting ibrutinib for CLL with a rapidly increasing WBC count. Due to concern for spontaneous tumor lysis syndrome and acute kidney injury, nephrology was consulted and recommended initiation of continuous veno-venous hemofiltration (CVVH). CVVH was used due to hemodynamic instability, given the patient’s mean arterial pressure (MAP) of 70 mmHg and significant anemia. The patient was transferred to the intensive care unit. He was started on rasburicase given his hyperuricemia (uric acid of 9 mg/dL). Ibrutinib was initiated two days after admission to treat his CLL. The patient eventually transitioned from CVVH to intermittent hemodialysis (IHD). Ibrutinib was briefly held due to a drop in the patient’s hemoglobin (6.3 g/dL) and the patient requiring a transfusion of one unit of packed red blood cells but was resumed after hemoglobin stabilized to 7.1 g/dL.

The patient’s clinical condition initially improved with therapy; however, he developed a recurrent pericardial effusion and was recommended to have a pericardial window performed to manage this. The patient declined surgical intervention and expressed his wish to transition to comfort care measures. He passed away one day later in the hospital.

## Discussion

Although CLL is considered incurable, treatment depends on the presence of disease-related complications [2,4]. The National Comprehensive Cancer Network (NCCN) outlines multiple treatment regimens for CLL, including acalabrutinib ± obinutuzumab, ibrutinib, venetoclax, or zanubrutinib [5]. Patients with CLL receiving these therapies are at risk of developing tumor lysis syndrome (TLS) [6,7].

TLS is an oncological emergency resulting in electrolyte derangements due to tumor cell death, which can lead to life-threatening arrhythmias and acute renal failure [3]. The risk factors of TLS include hematological malignancies with high proliferative rates, high tumor burdens, preexisting elevated uric acid levels, cytotoxic therapy, poor hydration, and malignancies that have infiltrated the kidneys [3]. TLS is a condition associated with cytotoxic chemotherapy since the objective of these regimens is to kill malignant cells, thereby leading to the release of electrolytes upon cancer cell death [3]. The diagnosis of TLS can be made using the Cairo-Bishop classification (Table 2). To make the diagnosis of TLS based on this classification, the patient needs to have at least two of the laboratory criteria and one of the clinical criteria [3]. Our patient had two of the laboratory criteria (hyperphosphatemia and hyperuricemia) and one of the clinical criteria (acute kidney injury), therefore meeting the Cairo-Bishop classification for TLS.

TLS can also occur spontaneously without the initiation of cytotoxic chemotherapy, as seen in our patient. Symptoms manifest as life-threatening organ dysfunction, such as acute renal failure and cardiac arrhythmia [8]. While an exact incidence is not known, spontaneous TLS is understood to rarely occur, although cases have been reported [9,10]. One study showed that three out of 33 patients with non-Hodgkin’s lymphoma (NHL) had marked hyperuricemia and acute kidney injury before receiving chemotherapy [11], which was also seen in our patient. One laboratory value that may differ between spontaneous TLS and TLS due to chemotherapy is the phosphorus level [12]. High cell turnover of cells should lead to the release of uric acid and phosphorus, among other electrolytes. In spontaneous TLS, the tumor is actively metabolizing due to rapid turnover and reuptakes the released phosphorus to use in its metabolism [12]. However, if chemotherapy has been initiated, the tumors are subject to cytotoxic exposure with the hopes of tumor death. As such, in this scenario, there is no reuptake, or at the very least reduced reuptake, of phosphorus, resulting in TLS with hyperphosphatemia [12].

Both spontaneous TLS and TLS as a consequence of chemotherapy can cause electrolyte derangements that can ultimately lead to organ failure. For our patient, despite the absence of cytotoxic chemotherapy and a known stable malignancy, it was imperative to consider TLS given the patient’s electrolyte abnormalities and the potential to cause renal failure, which may have been in progress given his worsening kidney functions.

Since both spontaneous TLS and TLS as a consequence of chemotherapy will lead to similar electrolyte disarrangements, the goal of management for both situations is to correct metabolic derangements. Rasburicase and allopurinol are the hypouricemic agents of choice to control elevated uric acid levels and prevent renal failure [13]. Hyperkalemia, which can lead to lethal cardiac arrhythmias, can be addressed by limiting potassium and phosphate intake [14]. Glucose with insulin or beta agonists as well as calcium gluconate can also be used [14]. If these methods are insufficient, hemodialysis and hemofiltration would be needed to lower potassium levels since this would likely occur with damage to the kidneys, as seen with our patient [14]. If the patient is showing signs of symptomatic hypocalcemia, calcium can be given [14]. If hyperphosphatemia is present, calcium should be held until hyperphosphatemia normalizes to prevent calcium-phosphate precipitation, unless the patient has severe symptoms such as cardiac arrhythmias [14]. Hyperphosphatemia can be addressed with aggressive IV hydration but will often cause acute kidney injury and can require hemodialysis or hemofiltration to adequately lower phosphorus levels, as seen in our patient [15].

## Conclusions

We present a case of spontaneous TLS, an oncologic emergency, that occurred in a patient with a stable malignancy despite not being treated with cytotoxic chemotherapies, resulting in an imbalance of electrolytes and increasing the risk of life-threatening arrhythmias and acute renal failure. For patients with a known malignancy but not receiving chemotherapy at the time and presenting with signs of an acute kidney injury, hyperphosphatemia, hyperuricemia, hyperkalemia, and hypocalcemia, it is important to keep spontaneous TLS as part of the differential diagnosis. Aggressive intravenous hydration, rasburicase, and limiting potassium and phosphate intake will be needed to control the electrolyte imbalance, and if these prove insufficient, timely initiation of renal replacement therapy is imperative.
